# Cell Type Specific Representation of Vibro-tactile Stimuli in the Mouse Primary Somatosensory Cortex

**DOI:** 10.3389/fncir.2018.00109

**Published:** 2018-12-20

**Authors:** Ayako Hayashi, Takashi Yoshida, Kenichi Ohki

**Affiliations:** ^1^ Department of Molecular Physiology, Graduate School of Medical Sciences, Kyushu University, Fukuoka, Japan; ^2^ Department of Dermatology, Graduate School of Medical Sciences, Kyushu University, Fukuoka, Japan; ^3^ Department of Physiology, Graduate School of Medicine, The University of Tokyo, Tokyo, Japan; ^4^ CREST, Japan Agency for Medical Research and Development (AMED), Tokyo, Japan; ^5^ International Research Center for Neurointelligence (WPI-IRCN), The University of Tokyo Institutes for Advanced Studies (UTIAS), Tokyo, Japan

**Keywords:** somatosensory cortex, vibro-tactile sensation, inhibitory cells, functional subnetworks, two-photon imaging

## Abstract

Although the processing of whisker deflections in the barrel area of the rodent primary somatosensory cortex (S1) has been studied extensively, how cutaneous vibro-tactile stimuli are processed in the rodent S1 outside the barrel area has not been fully examined. Particularly, the cell-type specific representation of multiple vibration frequencies in genetically identified inhibitory cells in the S1 has not been examined. Using two-photon calcium imaging, we examined the responses to vibration stimuli of excitatory and inhibitory neurons in the S1 hind limb area of male and female mice. The excitatory cells showed relatively sharp selectivity to vibration stimuli, whereas the inhibitory cells exhibited less selectivity. The excitatory and inhibitory cells with different preferred stimuli were intermingled in a “salt and pepper” manner. Furthermore, the noise correlation tended to be especially strong in excitatory-inhibitory and inhibitory-inhibitory cell pairs that have similar stimulus selectivity. These results suggest that excitatory cells tend to represent specific stimulus information and work together with similarly tuned inhibitory cells as a functionally connected network.

## Introduction

Vibro-tactile stimuli applied to the skin evoke various sensations. Depending on the vibratory frequency, various cutaneous mechanoreceptors are activated. Merkel cells are tuned to the “texture” of the object (or vibration frequency of 0.4–2.0 Hz), Meissner’s corpuscles are tuned to “flutter” (2–40 Hz), and Pacinian corpuscles are tuned to “vibration” (40–500 Hz) (McGlone and Reilly, 2010; Abraira and Ginty, 2013). The signals from these receptors are projected to the S1 via the thalamus (rats: Angel and Clarke, 1975; cats: Brown et al., 1980; monkeys: Jones, 1983; Rausell and Jones, 1991; Friedman et al., 2004). Thus, S1 is crucial for vibro-tactile perception (monkeys: Paul et al., 1972; Sur et al., 1981; Pons et al., 1987; Iwamura et al., 1993; Burton et al., 1995; Zainos et al., 1997; Romo et al., 2000; Yau et al., 2013; mice: O’Connor et al., 2010; Sachidhanandam et al., 2013).

The vibro-tactile sense has been extensively studied in the rodent barrel cortex (de Kock et al., 2007; Gentet et al., 2010; Gerdjikov et al., 2010; O’Connor et al., 2010; Crochet et al., 2011; Adibi et al., 2012; Clancy et al., 2015). The neurons in each barrel are most strongly driven by their principal whisker and more weakly by the surrounding whiskers (Simons, 1978). However, with advances in imaging techniques, the whisker selectivity of individual neurons within a barrel has been reported to be “salt and pepper”-like, that is, more heterogeneous than formerly imagined (Sato et al., 2007; Clancy et al., 2015). It has also been reported that the neurons in the barrel code the velocity of passive whisker vibrations (Pinto et al., 2000; Arabzadeh et al., 2004; Gerdjikov et al., 2010).

However, how neurons in the rodent S1 excluding the barrel area represent Vibro-tactile stimuli is not well understood. Previous studies have reported responses to only a single frequency vibration (Winship and Murphy, 2008) and simple touch stimulus (Tutunculer et al., 2006; Foffani et al., 2008; Storchi et al., 2012).

How vibro-tactile stimuli are represented by excitatory and inhibitory neurons also remains to be examined. The vast majority (~80%) of neurons are excitatory, and inhibitory cells are a minority (Rudy et al., 2011; DeFelipe et al., 2013; Petersen and Crochet, 2013). Excitatory neurons are sparsely connected to each other, whereas synaptic interactions between excitatory and fast spiking GABAergic inhibitory neurons are dense (Holmgren et al., 2003; Packer and Yuste, 2011; Avermann et al., 2012), which may cause the two cell types to have different response properties. In the barrel cortex, the inhibitory cells are more responsive than the excitatory cells (Gentet et al., 2012; Petersen and Crochet, 2013; Peron et al., 2015). Furthermore, the inhibitory cells are “broadly tuned” compared with the “narrowly tuned” excitatory cells in the mouse primary visual cortex (V1) (Sohya et al., 2007; Liu et al., 2009; Kerlin et al., 2010; Hofer et al., 2011; Atallah et al., 2012; but also see Runyan et al., 2010), olfactory bulb (Kato et al., 2013; Miyamichi et al., 2013), medial entorhinal cortex (Buetfering et al., 2014), and the hippocampus (Hu et al., 2014) but inconsistent in the mouse auditory cortex (Wehr and Zador, 2003; Wu et al., 2008; Sakata and Harris, 2009; Dorrn et al., 2010; Sun et al., 2010; Moore and Wehr, 2013; Li et al., 2015). In the S1 barrel area, the fast-spiking units, that is, the putative inhibitory cells, exhibit broad tunings compared to the regular-spiking units, that is, the putative excitatory cells (Bruno and Simons, 2002; Kida et al., 2005).

The broad tuning of inhibitory cells is proposed to reflect nonselective input integration from the surrounding excitatory cells (Kerlin et al., 2010; Bock et al., 2011; Hofer et al., 2011; Scholl et al., 2015). In contrast, recent studies have reported that the selective integration of inhibitory cells occurs in the mouse V1 after a learning process (Khan et al., 2018) and within the column structure in the ferret visual cortex (Wilson et al., 2017). Thus, how the inhibitory cells integrate inputs from the surrounding cells is an important topic for cortical processing and is still under debate. The integration patterns of the inhibitory cells are affected by functional architectures and connections in local circuits**,** which are variable across cortical areas and species (Harris and Mrsic-Flogel, 2013; Tremblay et al., 2016; Yavorska and Wehr, 2016). However, whether S1 inhibitory cells nonselectively integrate inputs from excitatory cells or selectively interact with specific subsets of excitatory cells remains to be determined. In brief, we address the following questions in our study. Are inhibitory cells broadly tuned compared to excitatory cells in the S1? If so, do inhibitory cells integrate inputs, or do inhibitory cells interact with specific subsets of excitatory cells?

## Materials and Methods

All experiments were carried out in accordance with the institutional animal welfare guidelines of the Animal Care and Use Committee of Kyushu University and approved by the Ethical Committee of Kyushu University.

### Animals

We obtained Gad2-IRES-Cre (Taniguchi et al., 2011, Jax stock #010802), Scnn1a-tg3-cre (Madisen et al., 2010, Jax stock #009613), and Ai14 mice (Madisen et al., 2010, Jax stock #007914) from the Jackson Laboratory. These mice were crossed to generate Gad2-Ai14 and Scnn1a-Ai14 mice. Both sexes were used. In Gad2-Ai14 mice, most cortical inhibitory neurons express tdTomato (Taniguchi et al., 2011). In Scnn1a-Ai14 mice, layer 4 neurons express tdTomato (Madisen et al., 2010).

### Vibrotactile Stimulation

A piezo device (3.2 × 0.6 cm in size, #Q220-A4-203YB, Piezo Systems, Inc., Woburn, MA, USA) controlled with an analog output board (PowerLab, ADInstruments or USB6003/6009, National Instruments), an amplifier (T-HVA03, TURTLE Industry, Japan), and custom written programs were used to apply vibro-tactile stimulation to the mouse’s right hind limb. The fur of the right hind limb skin was removed using hair remover lotion. The piezo device was gently placed on the surface of the extended right hind limb. We were careful to ensure that the piezo device gently touched the hind limb, rather than pressed against it. Each stimulus trial started with a blank period of 5 s, and then the piezo device vibrated for 4 s, followed by another blank period of 16 s before the next trial. The vibration frequency was changed in each trial and controlled by the voltage change applied to the piezo device (80 V and 2–200 Hz). Displacements of the piezo device were measured by a laser displacement meter in our stimulation setup, but without a mouse. The displacements were 1.7 × 10^2^, 1.7 × 10^2^, 1.7 × 10^2^, 1.7 × 10^2^, 1.7 × 10^2^, 1.9 × 10^2^, and 3.5 × 10^2^ μm for 2, 5, 10, 20, 50, 100, and 200 Hz, respectively. Thus, we were aware that we did not simply monitor “frequency tunings” especially at higher frequencies with our stimulation set. The vibration frequency parameters were 2, 5, 10, 20, 50, and 100 Hz for 6 mice and 2, 5, 10, 20, 50, 100, and 200 Hz for 5 mice. We found that the results tended to be similar between the two groups. Thus, we combined the data of the 11 mice. In a single imaging session, the vibration frequencies were delivered in a pseudorandom order in which each frequency appeared every 6 or 7 trials. Each frequency was repeated 16 times (a total of 96 trials with 6 frequency parameters in 6 mice and 112 trials with 7 frequency parameters in 5 mice).

### Intrinsic Signal Imaging

The mice were anesthetized with isoflurane (3.0% for induction, 1.5% for surgery, and 1.0% for imaging, Escain®, Mylan, Cecil Township, PA, USA). After a subcutaneous injection of lidocaine (2% Lidocaine Injection, Nagase Medicals Co., Ltd., Itami, Japan) was administered for local anesthesia, a sagittal incision was performed on the scalp. This incision was opened and tugged sideways to expose the skull. A custom-made metal head plate was attached to the skull using quick self-curing acrylic resin (ADFA, Shofu Inc., Kyoto, Japan). A drop of artificial cerebrospinal fluid (a simplified version) [ACSF; 150 mM NaCl, 2.5 mM KCl, and 10 mM HEPES (pH 7.4)] was placed and sealed with a glass cover slip to keep the skull moist and transparent. The mouse was placed on a heating pad to maintain its body temperature at 37°C. On the right hind limb, the piezo device was placed for stimulation, and the stimulation was applied 10 times. The skull was illuminated by LED light with a peak wavelength of 640 nm. The cortical images were obtained under a microscope (Me600, Nikon, Japan) with a 4× objective (Plan Apo, Nikon) and recorded at 5 Hz using a CCD camera (Adimec-1000, Adimec, Eindhoven, the Netherlands). The CCD camera was controlled by an Imager 3001 Laboratory Interface (Optical Imaging Ltd., Rehovot, Israel). After the imaging, the head plate was removed, and the skin was sutured. The mice were allowed to recover for the following virus injection.

### Virus Injection

The mice were anesthetized with isoflurane, and the suture was reopened. Using a dental drill, a small hole was created on the skull at the S1 hind limb area, which was identified by the previously performed intrinsic imaging. AAV2/1-syn-GCaMP6s (~10^12^ genomes per milliliter, vector core; University of Pennsylvania, Philadelphia, PA, USA. Chen et al., 2013) was slowly injected (0.05 μl/min, 10 min) though a glass pipette (20–30 μm tip diameter) inserted at approximately 400 μm below the cortical surface. The viral titer was adjusted to minimize overexpression of GCaMP6s (Sachidhanandam et al., 2013). After the injection, the suture was closed again, and the mouse was allowed to recover with great care.

### *In vivo* Two-Photon Calcium Imaging

The mice were kept for at least 2 weeks after the virus injection to ensure GCaMP6s expression. The mice were anesthetized with isoflurane (3.0% for induction, 1.5% for surgery, and 1.0% for imaging), and the metal plate for head fixation was attached to the skull as described above. We also administered an intraperitoneal injection of dexamethasone (4 mg/kg, Dexart®, Fujiseiyakukougyou Co., Ltd., Toyama, Japan) to prevent inflammation, atropine (0.22 mg/kg, atropine sulfate, FUSO Pharmaceutical Industries, Ltd., Osaka, Japan) to secure the airway, and mannitol to prevent cortical edema. Craniotomy was performed above the S1 hind limb region, and a small opening (3.5 mm) was created on the skull. The opening was filled with ACSF and sealed with a glass cover slip. We used a two-photon microscope (Olympus FVMPE-RS) for the calcium imaging. The excitation light was focused with a 25× objective (XLPlan N, Olympus). GCaMP6s was excited at a 920 nm wavelength, and tdTomato at a 1120 nm wavelength (Insight Deep See, Spectra-Physics, Santa Clara, CA, USA). The images were obtained using Olympus FV software. A square region of approximately 390 × 390 μm was imaged at 512 × 512 pixels and a 30 Hz frame rate using a resonant scanner. The imaging depth ranged from 160 to 340 μm below the cortical surface (*n* = 26 planes from 11 mice). The boundary of layers 2/3 and 4 was estimated from the two-photon volume images of Scnn1a-Ai14 transgenic mice. Scnn1a-Ai14 mice express tdTomato in layer 4 (Madisen et al., 2010, Supplementary Figure 1). We consider our data to be from layer 2/3.

### Data Analysis

The images were analyzed using MATLAB (Mathworks, Natick, MA, USA). For the optical imaging experiments, the baseline signal (S) of each trial was the averaged intrinsic signals during 1 s before each stimulus onset. The single-trial responses from which the baseline signals were subtracted were divided by the baseline signals to obtain the intrinsic signal ratio changes (dS/S). To obtain the response map, the dS/S was averaged per second from the 2 s before the stimulus onset to 13 s after the stimulus onset and averaged across trials.

For the two-photon data, the imaged frames were realigned by maximizing the correlation between the frames. For cell-based analysis, the images were averaged across all frames and filtered to remove the low spatial frequency component and enhance the ring-like structure of the GCaMP-expressed soma (Gaussian filter, sigma = 3–5 pixels roughly corresponding to the thickness of the ring). In the time-averaged image, the cell locations were identified by nuclei where the GCaMP signal did not localize, and the nuclei centers were manually selected. Within the radius of the soma, 5–8 pixels from the nucleus center, bright pixels around the nucleus (>1 standard deviation + mean of all pixels in the image) were detected and defined as the region of interest (ROI) in the individual cells. The ROIs were manually corrected by visual inspection. The time courses of the individual cells were extracted by averaging the pixel values within the ROI. Slow drifts of the baseline signal over minutes were removed by a low-cut filter (Gaussian, cutoff 100 s), and high frequency noises were removed by a high-cut filter (5th order Savitzky-Golay filter for 31 frame points corresponding to approximately one second). To minimize neuropil signal contamination (i.e., out of focus signal contamination), the time courses of the neuropil signal obtained from the surrounding, ring-shape regions of the cell contours were subtracted from time course of each neuron after multiplying it by a scaling factor (Kerlin et al., 2010). The scaling factor was set at 1.0. This value is slightly higher than that in a previous report (e.g., 0.7) (Chen et al., 2013). It was set slightly higher to minimize the effects of the neuropil signal contamination on the analyses (see below), specifically the noise and signal correlation analyses. After removing the neuropil signal, the time course was used to obtain the signal change (mean fluorescence change normalized to the baseline, dF/F) in the response to each stimulus frequency. In the following analyses, the time-averaged responses during the stimulation and 5 s prestimulus periods (baseline) were used.

Significant responses were identified by comparing the prestimulus and poststimulus signals during the 16 repeated trials of each vibro-tactile stimulus frequency (*P* < 0.05, Wilcoxon’s signed-rank test and trial-averaged evoked response amplitude >3%). Responsive cells were defined as cells with a significant response to at least one stimulus frequency. Using these data, the percentages of responsive cells were determined. For each responsive cell, the stimulus frequency that provoked the largest response (i.e., the stimulus frequency that initiated the largest dF/F) was defined as the best frequency. The number of responded stimuli refers to the number of vibration frequencies (2–100 Hz) that evoked a significant response in each cell. The selectivity index (SI) was calculated as follows in each responsive cell across stimulus frequencies: SI = (largest response − smallest response)/(largest response + smallest response). The noise correlation and signal correlation were calculated for all possible responsive cell pairs in each imaged plane. For the noise correlation during stimulation, single trial-responses from which the trial-averaged response was subtracted were transformed to *z*-scores in each stimulus frequency. These *z*-scored responses were collected across frequencies in each cell. The noise correlation was defined as Pearson’s correlation of the *z*-scored responses between two cells. For the correlations of baseline activity, Ca^2+^ signal time courses during baseline periods were concatenated across stimuli and trials and were used for calculations of the correlation (Hofer et al., 2011). For the signal correlation analysis, a response pattern curve across frequencies was generated with the trial-averaged responses in each cell. Signal correlation was defined as Pearson’s correlation of the response pattern curves between two cells. In the signal correlation between a single excitatory cell and the average of excitatory cells, the selected single cell was excluded from the calculation of the average. For the inhibitory cells, all excitatory cells were used for the average of excitatory cells. Similar examination has been made in the previous studies (Kerlin et al., 2010; Scholl et al., 2015).

In the results for all cells (all cell comparisons, e.g., Figure 3A), the data of all responsive cells were collected across planes and animals. In the results for individual animals (across-animal comparison, e.g., Figure 3B), the data of all responsive cells were first collected for individual animals. The median (not the mean) of the collected data was computed for each animal and collected across animals. For correlation between noise correlations during stimuli and time course correlation during baseline (Figures 5E,F) and correlation between the noise and signal correlation (Figures 6E,F), Pearson’s correlation was computed in each plane. For the across-plane comparisons (Figures 5E,6E), the correlation coefficients of individual planes were collected across planes and across animals. For the across-animal comparisons (Figures 5F,6F), the mean of the correlation coefficients for each animal was computed and collected across animals.

### Statistical Analyses

All error bars shown in this article indicate standard error of mean unless otherwise stated. The statistical analyses were performed across both all responsive cells and animals for our results’ reliability. The significant level was set at 0.05. If statistical tests were repeated, *p* values were adjusted by the Bonferroni correction.

## Results

### Two-Photon Calcium Imaging of Gad2-Ai14 Transgenic Mice Transfected With GCaMP6s

We used Gad2-Ai14 transgenic mice to distinguish the inhibitory cells from the excitatory cells (Taniguchi et al., 2011). In Gad2-Ai14 transgenic mice, cortical GABAergic inhibitory neurons are genetically labeled by tdTomato, allowing us to discriminate the inhibitory cells from the excitatory cells. These mice were injected with AAV2/1-Syn-GCaMP6s in the S1 hind limb area. The AAV2/1-Syn-GCaMP6s injection causes both excitatory and inhibitory cells around the injected area to express GCaMP6s (Chen et al., 2013), enabling us to record their neuronal responses as changes in fluorescence signal.

**Figure 1:**
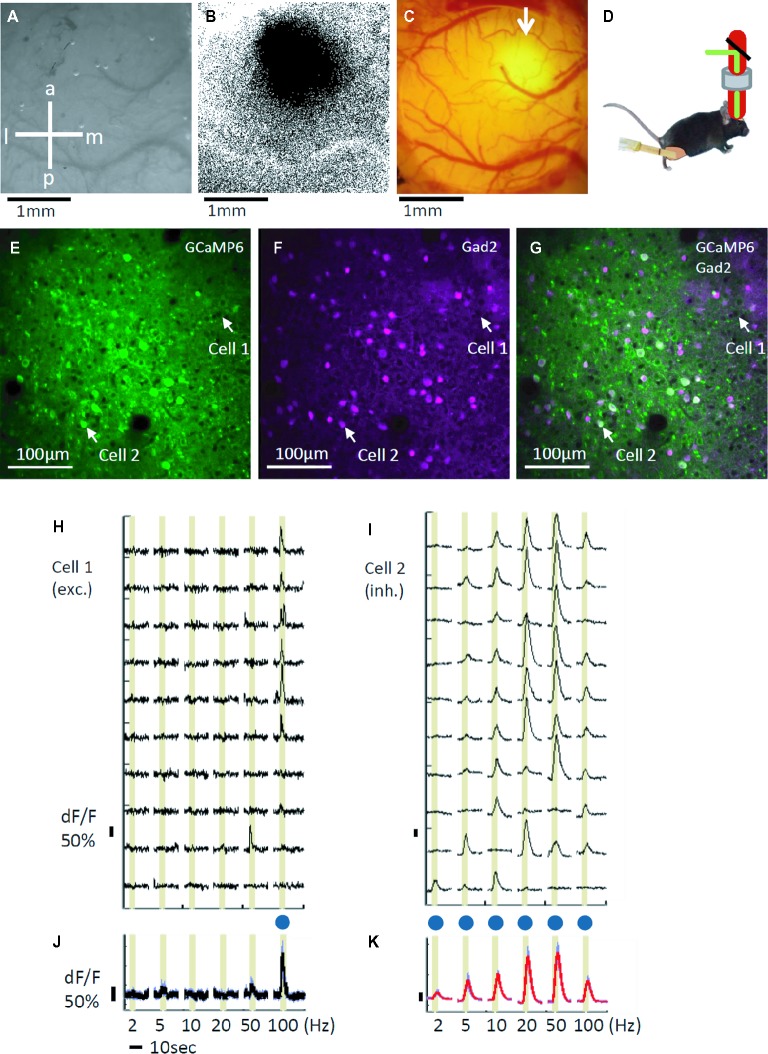
*In vivo* calcium imaging by two-photon microscopy. **(A)** Image of the left primary somatosensory area (S1) surface. The skull is still intact and kept moist for transparency. a: anterior, p: posterior, l: lateral, and m: medial. **(B)** Response map to a 100 Hz vibration stimulus recorded by intrinsic optical imaging; the darker area is the identified hind limb area in S1. **(C)** GCaMP6s expression in the hind limb area (white arrow). Craniotomy was performed approximately 3 weeks after the virus injection. The background reddish signal is due to tdTomato expressed in gad2-positive cells. **(D)** Scheme of the vibro-tactile stimulation during imaging. Piezo equipment was placed on the right hind limb to apply the vibration stimuli. Animals were anaesthetized with isoflurane during imaging. **(E)** Two photon imaging of GCaMP signals. Imaging is performed at a 920 nm wavelength. The white arrows indicate excitatory (cell 1) and inhibitory (cell 2) cells. **(F)** Two photon imaging of gad2-positive inhibitory cells at the same plane as shown in **(E)**. tdTomato was expressed in gad2-positive cells, and the image was obtained at a 1120 nm wavelength. The white arrows indicate excitatory (cell 1) and inhibitory (cell 2) cells. **(G)** The merged image of GCaMP (**E**, green) and the gad2 (**F**, magenta) images. **(H,I)** Examples of an excitatory **(H)** and an inhibitory **(I)** cell’s dF/F traces. The cell positions are indicated with white arrows **(E–G)**. Of the 16 performed trials, 10 trials’ traces are shown. Stimulus periods are indicated by yellow shadings. **(J,K)** Averaged traces during the 16 performed trials for the excitatory (**J**, cell 1) and the inhibitory (**K**, cell 2) cells. Blue dots indicate significant responses.

First, intrinsic optical imaging was performed to identify the S1 hind limb area (Figures 1A,B). A piezo device was placed on the right hind limb to provide the vibration stimuli, and the hind limb area was identified in the left S1 (Figure 1B). The identified area was injected with AAV2/1-Syn-GCaMP6s, and after two to three weeks, GCaMP6s expression was observed (Figure 1C). Then, we performed two-photon calcium imaging of the GCaMP6s expressing neurons in layer 2/3 of the S1 hind limb area. During imaging, the mice were anesthetized with isoflurane, and vibro-tactile stimuli were applied to the right hind limb (Figure 1D). GCaMP6s expression was confirmed in both tdTomato-positive and tdTomato-negative cells with two-photon microscopy (Figures 1E–G). Hereafter, we call the tdTomato-positive cells inhibitory cells (Inh) and the tdTomato-negative cells excitatory (Exc) cells, although not all inhibitory cells are labeled by tdTomato (Taniguchi et al., 2011). We recorded the responses of 2691 excitatory and 696 inhibitory cells in our two-photon imaging (excitatory: ~79%, inhibitory: ~21%; total of 26 planes in 11 mice). The ratio of excitatory and inhibitory cells is consistent with previous reports (Rudy et al., 2011; DeFelipe et al., 2013; Petersen and Crochet, 2013).

We found that both excitatory and inhibitory cells responded to the vibration stimuli. Figure 1H shows examples of the response time course representative excitatory cell that demonstrates sharp stimulus selectivity and only significantly responds to the 100 Hz stimulation (also see Figure 1J for the average of response time courses for 16 trials). In Figure 1I, response time courses of a representative inhibitory cell that demonstrates relatively low selectivity and significantly responds to all 6 frequencies are shown (also see Figure 1K for the average of response time courses for 16 trials).

We also noticed a difference in the response time courses between the excitatory and the inhibitory cells (Supplementary Figure 2). This is probably due to the excitatory and the inhibitory cells, especially parvalbumin cells, having different firing patterns and/or different calcium binding proteins (Lee et al., 2000a,b; Kerlin et al., 2010; Eggermann et al., 2011). Similar differences in the response time courses between the excitatory and the inhibitory cells have been reported in the mouse visual cortex (Sohya et al., 2007; Kerlin et al., 2010).

### Low Stimulus Selectivity in Inhibitory Cells

**Figure 2:**
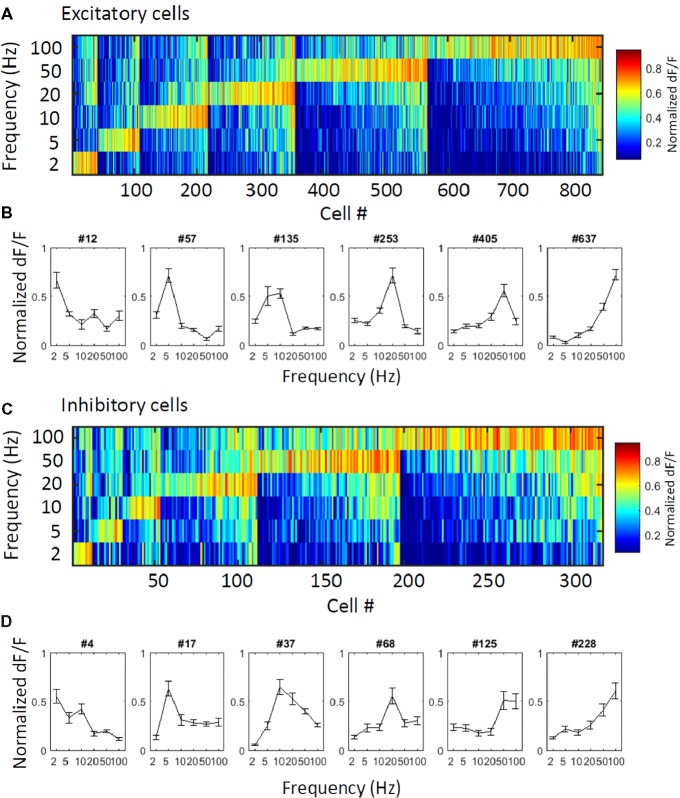
Overall view of the responses of the excitatory and inhibitory cells. **(A,C)** Normalized responses of excitatory **(A)** and inhibitory **(C)** responsive cells are depicted in color codes. To obtain the normalized responses in each cell, trial-averaged time course was normalized by its maximum value and then averaged during each stimulation period. Cells were sorted by their best frequencies (i.e., the stimulus that evoked the largest response from a neuron). All stimulus frequencies (2–100 Hz) are covered, and the number of responsive cells increases as the stimulus frequency increases in both cell types. **(B,D)** Response pattern curves of representative excitatory **(B)** and inhibitory cells **(D)**. Inhibitory cells show relatively less selectivity.

First, to obtain an overall perspective of how excitatory and inhibitory cells respond to vibration stimuli, we plotted response patterns of the excitatory and inhibitory cells. All responsive excitatory cells (*N* = 847 cells) and inhibitory cells (*N* = 319 cells) are sorted by their best frequencies (for definition, see Methods), and their normalized response magnitudes (ΔF/F) are represented in color codes (Figure 2A for excitatory cells and Figure 2C for inhibitory cells). Figures 2B,D shows the response patterns of representative excitatory and inhibitory cells, respectively. All six applied vibro-tactile frequencies were able to evoke responses in a proportion of cells of both cell types, and the best response frequencies of the excitatory and inhibitory cells were distributed in all six frequencies. A larger proportion of both excitatory and inhibitory cells strongly responded to the higher frequencies compared to the lower frequencies. Upon a closer examination, we observed that in the inhibitory cells, warm colors tend to spread over multiple stimulus frequencies in a larger proportion of cells, showing that the inhibitory cells are less selective to the stimuli. This finding is also depicted in the response patterns of representative cells (Figures 2B,D).

**Figure 3:**
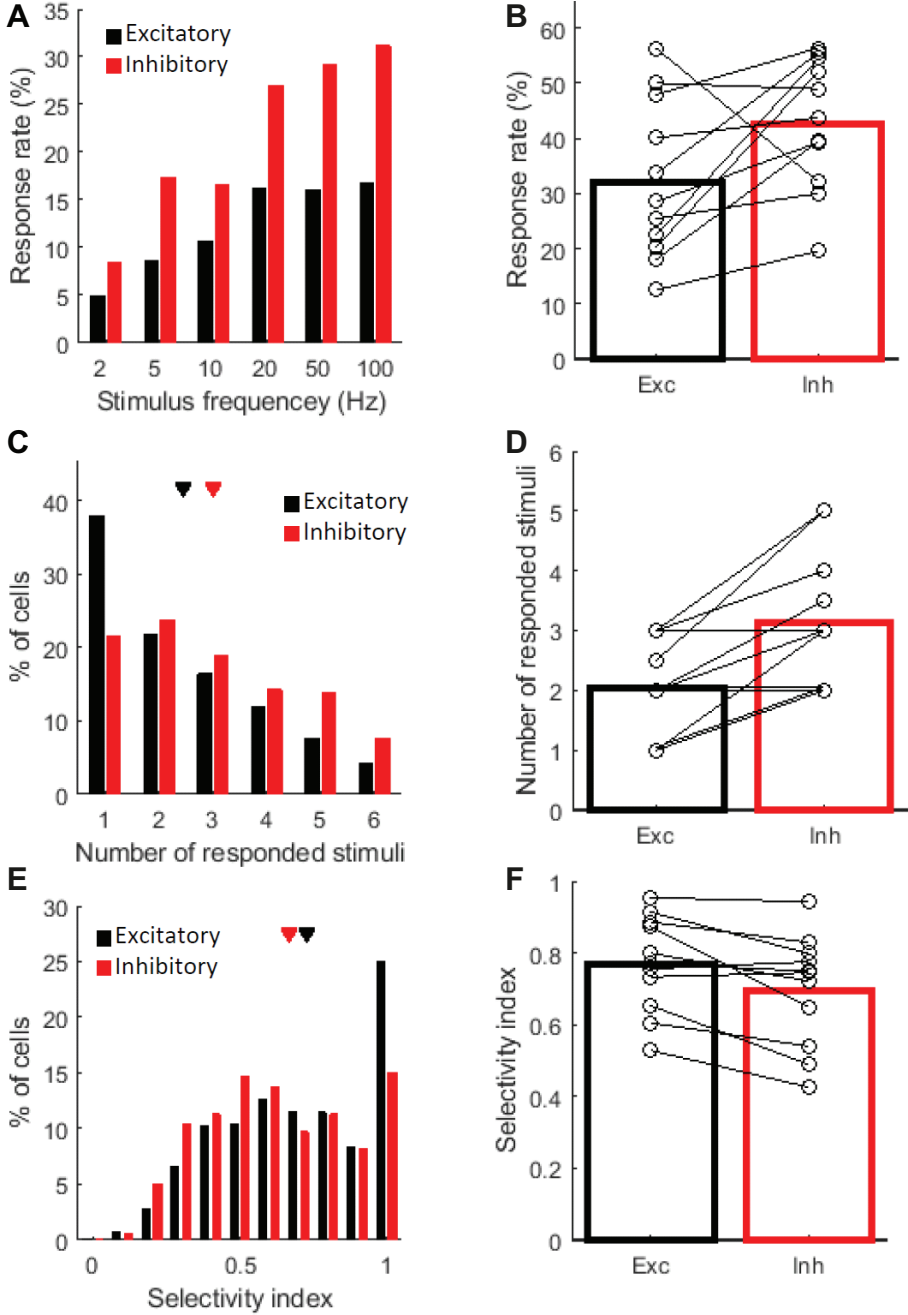
Response properties of excitatory and inhibitory cells. The following three response properties are shown: the percentage of responsive cells **(A,B)**, the number of responded stimuli **(C,D)**, and the selectivity index **(E,F)**. Black indicates excitatory cells and red indicates inhibitory cells. Each figure on the left **(A,C,E)** shows the distribution of all responsive cells observed across animals; 847 (out of 2691 cells) excitatory and 319 (out of 696) inhibitory cells were responsive for at least one stimulus frequency in 11 mice. Each figure on the right **(B,D,F)** shows the averaged values of the 11 mice, and each open circle indicates the median of one mouse. **(A)** Percentages of responsive cells for each stimulus frequency are shown. The values were calculated in each cell type. **(B)** Percentage of responsive cells at least for one stimulus frequency in each cell type. Exc: 32 ± 4.3% (mean ± SE) and Inh: 43 ± 3.6%. *N* = 11 mice. **(C–F)** Data from responsive cells were only included. **(C)** Exc: 2.5 ± 0.05 and Inh: 3.0 ± 0.09. **(D)** Exc: 2.0 ± 0.24 and Inh: 3.1 ± 0.34. **(E)** Exc: 0.73 ± 0.01 and Inh: 0.67 ± 0.01. **(F)** Exc: 0.77 ± 0.04 and Inh: 0.70 ± 0.05. **(C,E)** Triangles indicate the mean value for each cell type.

Then, to confirm the above interpretation, we computed several indexes related to the response properties and compared these indexes between the cell types. First, we looked at the proportion of responsive cells in each cell type population. The proportion of responsive cells tended to be higher among the inhibitory cells. The proportion of responsive cells in the inhibitory cell was significantly higher in the all cell comparison (Figure 3A; *χ* ^2^(1) = 11.9, *P* = 0.0033 for 2 Hz; *χ* ^2^(1) = 44.6, *P* = 1.5 × 10^−10^ for 5 Hz; *χ* ^2^(1) = 17.1, *P* = 2.2 × 10^−4^ for 10 Hz; *χ* ^2^(1) = 50.0, *P* = 3.3 × 10^−10^ for 20 Hz; *χ* ^2^(1) = 61.1, *P* = 3.2 × 10^−14^ for 50 Hz; and *χ* ^2^(1) = 72.2, *P* < 7.0 × 10^−16^ for 100 Hz by the *χ* ^2^ test at each frequency with the Bonferroni correction), and in the across-animal comparison (Figure 3B, *n* = 11 mice, *P* = 0.042 by the signed-rank test, Exc: 32 ± 4.3% (mean ± SE) and Inh: 43 ± 3.6%, percentage of cells that showed a significant response to at least one frequency). Next, we compared the stimulus selectivity. The inhibitory cells responded to more stimulus frequencies than the excitatory cells (Figure 3C; Exc: 2.5 ± 0.05 and Inh: 3.0 ± 0.09, *P* = 8.2 × 10^−6^ by the Kolmogorov–Smirnov (KS) test in the all cell comparison. Figure 3D; Exc: 2.0 ± 0.24 and Inh: 3.1 ± 0.34 stimulus frequencies. *P* = 0.0078 by the signed-rank test in the across-animal comparison). This finding is also reflected in the lower selectivity index of the inhibitory cells (Figure 3E; Exc: 0.73 ± 0.01 and Inh: 0.67 ± 0.01. *P* = 0.00095 by the KS test in the all cell comparison. Figure 3F; Exc: 0.77 ± 0.04 and Inh: 0.70 ± 0.05. *P* = 0.0098 by the signed-rank test in the across-animal comparison. See Methods for a definition of the selectivity index). Thus, the excitatory cells were presumed to encode a fewer number of frequencies, whereas the inhibitory cells showed relatively lower selectivity.

### Inhibitory Cells Tend to Share Stimulus Selectivity With Some Exceptions

**Figure 4:**
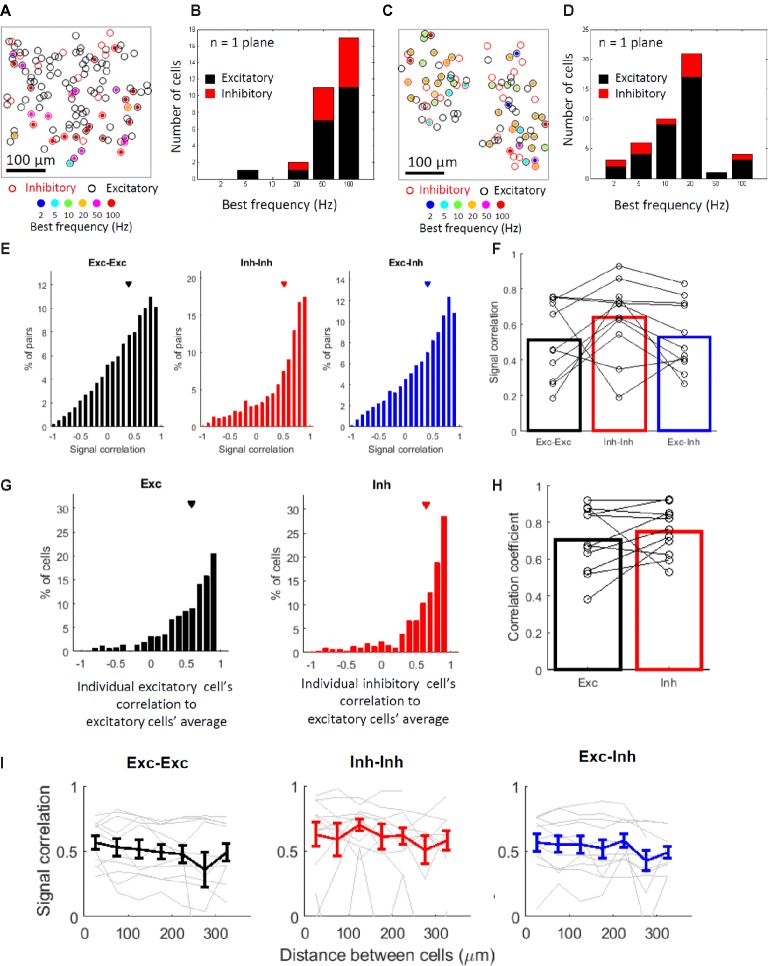
Inhibitory cells tend to share stimulus selectivity with some exceptions. **(A,C)** Representative planes depicting the responding cells’ best stimulus frequencies in color codes. Black and red open circles indicate locations of excitatory and inhibitory cells, respectively. Filled-in circles indicate significantly responsive cells, and their color indicates their best frequencies. A square region (390 μm each side) is shown. **(B,D)** Histograms of the best stimulus frequencies for the representative plane shown in **(A,C)**. Black and red bars indicate excitatory and inhibitory cells, respectively. **(E)** Distributions of all responsive cell pairs’ signal correlation from all imaged planes. In **(E,F,I)** black indicates excitatory cell pairs (Exc-Exc), red indicates inhibitory cell pairs (Inh-Inh), and blue indicates excitatory and inhibitory cell pairs (Exc-Inh). Exc-Exc: 0.39 ± 0.003 (mean ± SE, *n* = 17977 pairs), Inh-Inh: 0.51 ± 0.01 (*n* = 2168 pairs), and Exc-Inh: 0.40 ± 0.004 (*n* = 11752 pairs). Triangles indicate the mean values. **(F)** Mean signal correlation in 11 mice (bars); the open circles indicate the signal correlation medians in individual mice. Exc-Exc: 0.51 ± 0.07 (mean ± SE), Inh-Inh: 0.64 ± 0.06, and Exc-Inh: 0.53 ± 0.06. **(G)** Correlation between the averaged excitatory cells’ selectivity and individual excitatory (left, Exc) and inhibitory cells’ selectivity (right, Inh). Exc: 0.59 ± 0.01 (mean ± SE, *n*= 847 cells) and Inh: 0.65 ± 0.02 (*n* = 319 cells). Triangles indicate the mean value for each cell type. **(H)** Mean correlation coefficients in 11 mice (bars); the open circles indicate the correlation coefficient medians in individual mice. Exc: 0.70 ± 0.05 and Inh: 0.75 ± 0.04. **(I)** Signal correlation against cell pair distance. The results of excitatory-excitatory (left, black), inhibitory-inhibitory (middle, red), and excitatory-inhibitory (blue) pairs are shown. Gray lines in each panel indicate data from a single mouse (*N* = 11 mice per panel). Error bars indicate standard errors across animals.

Next, we explored the stimulus selectivity and best frequency of the vibro-tactile stimuli among neighboring neurons within an individual imaged plane (<390-micron range). Figures 4A,C shows examples of planes depicting the best frequencies (i.e., the frequency that evoked the largest response from a neuron) of the responsive cells in color codes. The spatial arrangement of the best frequencies seemed to be “salt and pepper” with no discernable clusters, and both cell types showed a wide range of best frequencies (Figures 4B,D). It also seems that subsets of cells, especially inhibitory cells, tend to share their best frequency within a single plane on average. This finding suggests that many inhibitory cells and a fraction of excitatory cells within several hundred microns may share response properties.

To confirm these observations, we first examined the similarity of the stimulus selectivity between two cells within a single plane (Figures 4E,F). We examined the similarity of response patterns by calculating the Pearson correlation of the response patterns between two cells (i.e., signal correlation, see Methods). Although the signal correlation tended to be positive in all cell pairs, it tended to be higher in the inhibitory cell pairs compared with that in the excitatory pairs and excitatory-inhibitory pairs. The higher signal correlation in the inhibitory cell pairs was statistically significant in all cell pair comparisons (Figure 4E; all cell pair comparisons: *P* = 1.5 × 10^−45^ for Exc-Exc vs. Inh-Inh pairs, *P* = 1.3 × 10^−4^ for Exc-Exc vs. Exc-Inh pairs, *P* = 5.8 × 10^−32^ for Inh-Inh vs. Exc-Inh by the KS test with the Bonferroni correction), although it was not significant in the across-animal comparisons (Figure 4F; Across-animal comparison, signal correlation for Exc-Exc: 0.51 ± 0.07, Inh-Inh: 0.64 ± 0.06, Exc-Inh: 0.53 ± 0.06. *P* = 0.44 in Exc-Exc vs. Inh-Inh pairs, *P* = 0.25 in Exc-Exc vs. Exc-Inh pairs, *P* = 0.62 in Inh-Inh vs. Exc-Inh pairs by the signed-rank test with the Bonferroni correction). This finding suggests that the stimulus selectivity of the inhibitory cell pairs tend to be more similar than those of the excitatory cell pairs. The excitatory-excitatory and excitatory-inhibitory cell pairs are also positively correlated, indicating that the local population including both cell types tends to share stimulus selectivity.

The aforementioned sharper stimulus selectivity of excitatory cells and lower selectivity of inhibitory cells suggest that the inhibitory cells integrate inputs from cells with a relatively wide range of best frequencies (Kerlin et al., 2010; Scholl et al., 2015). The tendency of excitatory and inhibitory cell (Exc-Inh) pairs to have similar stimulus selectivity implies that the inhibitory cells integrate inputs from neighboring excitatory cells and/or that there are shared inputs from other layers or regions into the local population of excitatory and inhibitory cells without a direct interaction between them. In both cases, if inhibitory cells integrate inputs, their response patterns should be similar to the average response patterns of the surrounding excitatory cells. To explore this hypothesis, we examined the correlation between the response patterns of the individual inhibitory cells and the averaged response patterns of the excitatory cells and compared the correlation between the two cell types (Figures 4G,H, Kerlin et al., 2010; Scholl et al., 2015). This correlation was positive and relatively high in the inhibitory cells (Figure 4G; all cell comparison, *R* = 0.59 ± 0.01 (mean ± SE) for Exc cells and 0.65 ± 0.02 for Inh cells. *P* = 0.0040 by the KS test. Figure 4H; across-animals comparison, *R* = 0.70 ± 0.05 for Exc cells and 0.75 ± 0.04 for Inh cells, *n* = 11 mice, *P* = 0.32 by the signed-rank test). However, notably, the response patterns of some inhibitory neurons were negatively correlated with the average of the response patterns of the surrounding excitatory cells (Figure 4G, right). This indicates that some individual inhibitory cells have different stimulus selectivity from that of the excitatory cells’ average and do not follow the average of the surrounding excitatory cells. These inhibitory cells may receive inputs from the excitatory cells whose selectivity are different from that of the excitatory cells’ average.

We also examined the spatial clustering of stimulus selectivity. We plotted the signal correlation against cell pair distances (Figure 4I). The signal correlation of any cell pair type remained virtually constant, independent of the distances between the cells ( *χ* ^2^(6) = 5.1, *P* = 0.99 for Exc-Exc; *χ* ^2^(6) = 9.2, *P* = 0.48 for Inh-Inh; *χ* ^2^(6) = 10.9, *P* = 0.27 for Exc-Inh; by the Friedman test with the Bonferroni correction). These results indicate that there is no obvious relationship in which closer cells share similar selectivity and that the spatial arrangement of the stimulus selectivity in the S1 hind limb area is “salt and pepper.”

### Inhibitory Cells Are More Active Together Than Excitatory Cells

**Figure 5:**
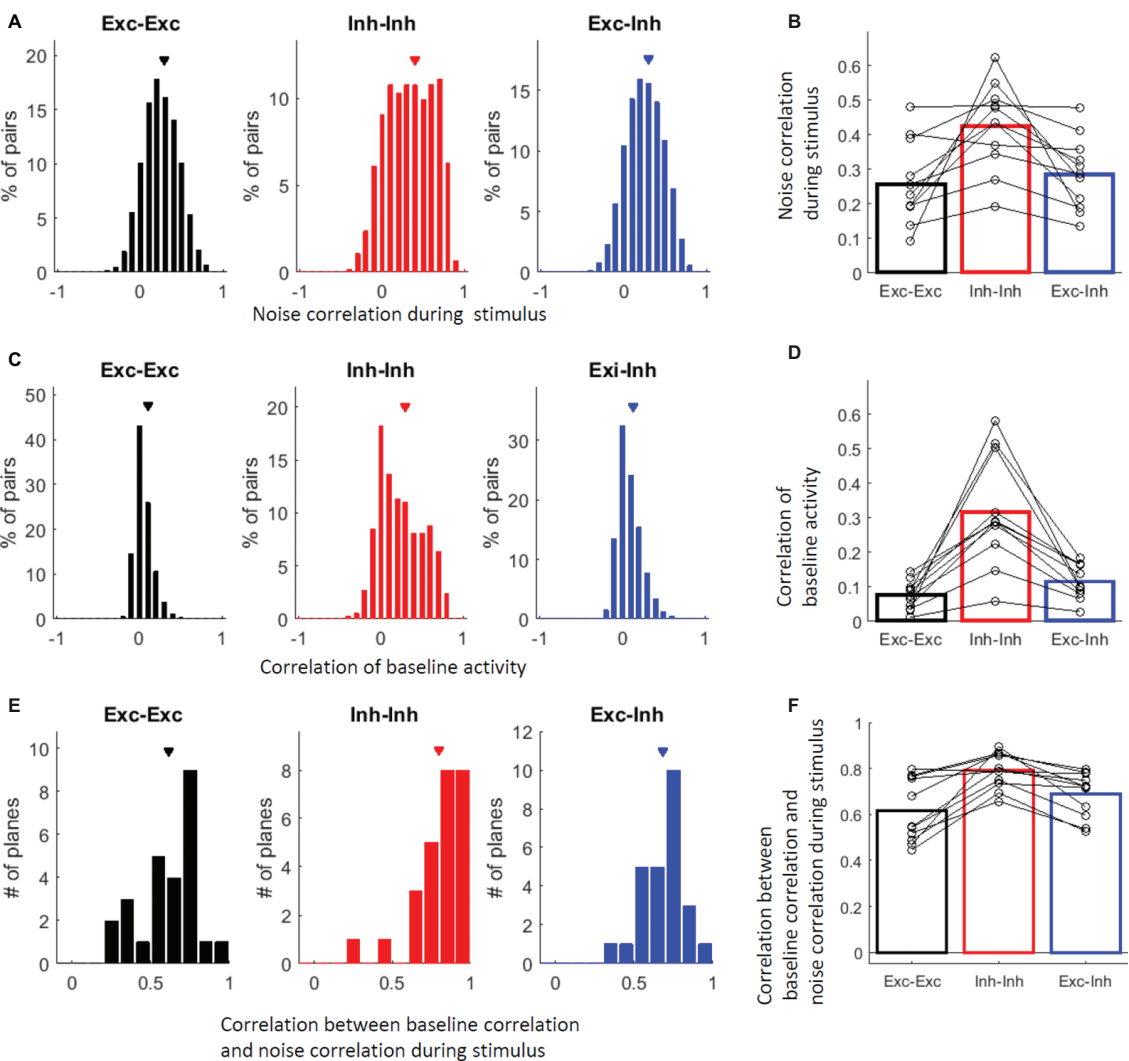
Inhibitory cells are more active together than excitatory cells. **(A)** Distribution of all responsive cell pairs’ noise correlations during the vibro-tactile stimulation. In **(A–F)** black indicates excitatory cell pairs, red indicates inhibitory cell pairs, and blue indicates excitatory and inhibitory cell pairs. Exc-Exc: excitatory pairs, Inh-Inh: inhibitory pairs, and Exc-Inh: excitatory and inhibitory pairs. Exc-Exc: 0.29 ± 0.002 (mean ± SE, *n* = 17977 pairs), Inh-Inh: 0.39 ± 0.01 (*n* = 2168 pairs), and Exc-Inh: 0.30 ± 0.002 (*n* = 11752 pairs). **(B)** Averaged noise correlations in 11 mice; the open circles indicate the noise correlation medians in individual mice. Exc-Exc: 0.26 ± 0.04, Inh-Inh: 0.43 ± 0.04, and Exc-Inh: 0.29 ± 0.03. **(C)** Distribution of all responsive cell pairs’ correlations of Ca^2+^ signal time course during baseline. Exc-Exc: 0.10 ± 0.0008, Inh-Inh: 0.29 ± 0.0057, and Exc-Inh: 0.13 ± 0.0013. **(D)** Averaged correlations of Ca^2+^ signal time courses during baseline in 11 mice; open circles indicate the noise correlation medians in individual mice. Exc-Exc: 0.0075 ± 0.012, Inh-Inh: 0.32 ± 0.048, and Exc-Inh: 0.12 ± 0.015. **(E,F)** Correlations between the time course correlation during baseline and the noise correlation during stimulation in all planes (*n* = 26 planes, **E**) and 11 mice **(F)**. The Pearson correlation between the noise correlations was computed in each plane and its distribution is shown in **(E)**. Exc-Exc: 0.62 ± 0.04, Inh-Inh: 0.80 ± 0.03, and Exc-Inh: 0.68 ± 0.03. **(F)** The correlations between the two noise correlations were averaged in each animal (open circles in **F**). Exc-Exc: 0.62 ± 0.04, Inh-Inh: 0.79 ± 0.02, and Exc-Inh: 0.69 ± 0.03. **(A,C,E)** Triangles indicate the mean values.

The relatively high similarity of the stimulus selectivity between the inhibitory cells suggests that inhibitory cells share inputs. Furthermore, inhibitory cell pairs are connected through gap junctions (Galarreta and Hestrin, 1999; Gibson et al., 1999; Tamás et al., 2000; Fukuda et al., 2006) and have been reported to show synchronous spontaneous activities (Hofer et al., 2011; Inoue et al., 2015; Karnani et al., 2016). These observations suggest that inhibitory cells may be more active together than excitatory cells. Therefore, we tested whether neurons tend to be active together when driven by vibro-tactile stimuli. Thus, we examined the noise correlations, that is, the correlation of the trial-to-trial activity fluctuation between two cells. The noise correlation was significantly higher in the inhibitory (Inh-Inh) cell pairs than in the excitatory (Exc-Exc) cell pairs and excitatory-inhibitory (Exc-Inh) cell pairs. This finding was observed across all cell pairs (Figure 5A; *P* = 1.1 × 10^−81^ for Exc-Exc vs. Inh-Inh, *P* = 5.6 × 10^−6^ for Exc-Exc vs. Exc-Inh, *P* = 4.5 × 10^−60^ for Inh-Inh vs. Exc-Inh; by the KS test with the Bonferroni correction) and animals (Figure 5B; Exc-Exc: 0.26 ± 0.04, Inh-Inh: 0.43 ± 0.04, Exc-Inh: 0.29 ± 0.03, *P* = 0.0088 for Exc-Exc vs. Inh-Inh, *P* = 0.30 for Exc-Exc vs. Exc-Inh, *P* = 0.0029 for Inh-Inh vs. Exc-Inh by the signed-rank test with the Bonferroni correction).

Cell-type specific correlated spontaneous activity has been reported (Hofer et al., 2011; Karnani et al., 2016), which is likely to reflect intrinsic network properties that are not driven by stimulation. To confirm cell-type specific correlated activity which is not driven by stimulation, we computed the correlation of Ca^2+^ signal time course during the baseline period (Hofer et al., 2011). The correlation of the baseline activity was also significantly higher in the inhibitory-inhibitory cell pairs than that in the excitatory-excitatory and excitatory-inhibitory cell pairs. This finding was observed across all cell pairs (Figure 5C, *P* = 1.3 × 10^−289^ for Exc-Exc vs. Inh-Inh, *P* = 2.2 × 10^−106^ for Exc-Exc vs. Exc-Inh, *P* = 5.5 × 10^−166^ for Inh-Inh vs. Exc-Inh; by the KS test with the Bonferroni correction) and animals (Figure 5D; Exc-Exc: 0.0075 ± 0.012, Inh-Inh: 0.32 ± 0.048, Exc-Inh: 0.12 ± 0.015, *P* = 0.0029 for Exc-Exc vs. Inh-Inh, *P* = 0.0029 for Exc-Exc vs. Exc-Inh, *P* = 0.0029 for Inh-Inh vs. Exc-Inh; by the signed-rank test with the Bonferroni correction).

Then, we examined whether the pairs of neurons that tended to be active together during baseline also tended to respond together during the application of the vibro-tactile stimuli. We found positive correlations between the noise correlation during the application of the vibro-tactile stimuli and the correlation during baseline activity, especially in inhibitory-inhibitory cell pairs. This finding was observed across all cell pairs (Figure 5E; Exc-Exc: 0.62 ± 0.04, Inh-Inh: 0.80 ± 0.03, Exc-Inh: 0.68 ± 0.03, *P* = 4.8 × 10^−5^ for Exc-Exc vs Inh-Inh, *P* = 5.7 × 10^−5^ for Exc-Exc vs Exc-Inh, *P* = 1.8 × 10^−3^ for Inh-Inh vs Exc-Inh; by the signed-rank test with the Bonferroni correction) and animals (Figure 5F; Exc-Exc: 0.62 ± 0.04, Inh-Inh: 0.79 ± 0.02, Exc-Inh: 0.69 ± 0.03, *P* = 0.0059 for Exc-Exc vs Inh-Inh, *P* = 0.029 for Exc-Exc vs Exc-Inh, *P* = 0.0029 for Inh-Inh vs Exc-Inh; by the signed-rank test with the Bonferroni correction). The higher correlations between the inhibitory-inhibitory cell pairs during baseline activity imply intrinsically shared inputs and/or dense connections between inhibitory cells, probably through gap junctions. The higher noise correlation during the application of the vibro-tactile stimuli and its high correlation to baseline activity imply that such connections are involved in activities during the application of the stimuli.

### Excitatory and Inhibitory Cells With Similar Stimulus Selectivity Are More Active Together Across Trials

**Figure 6:**
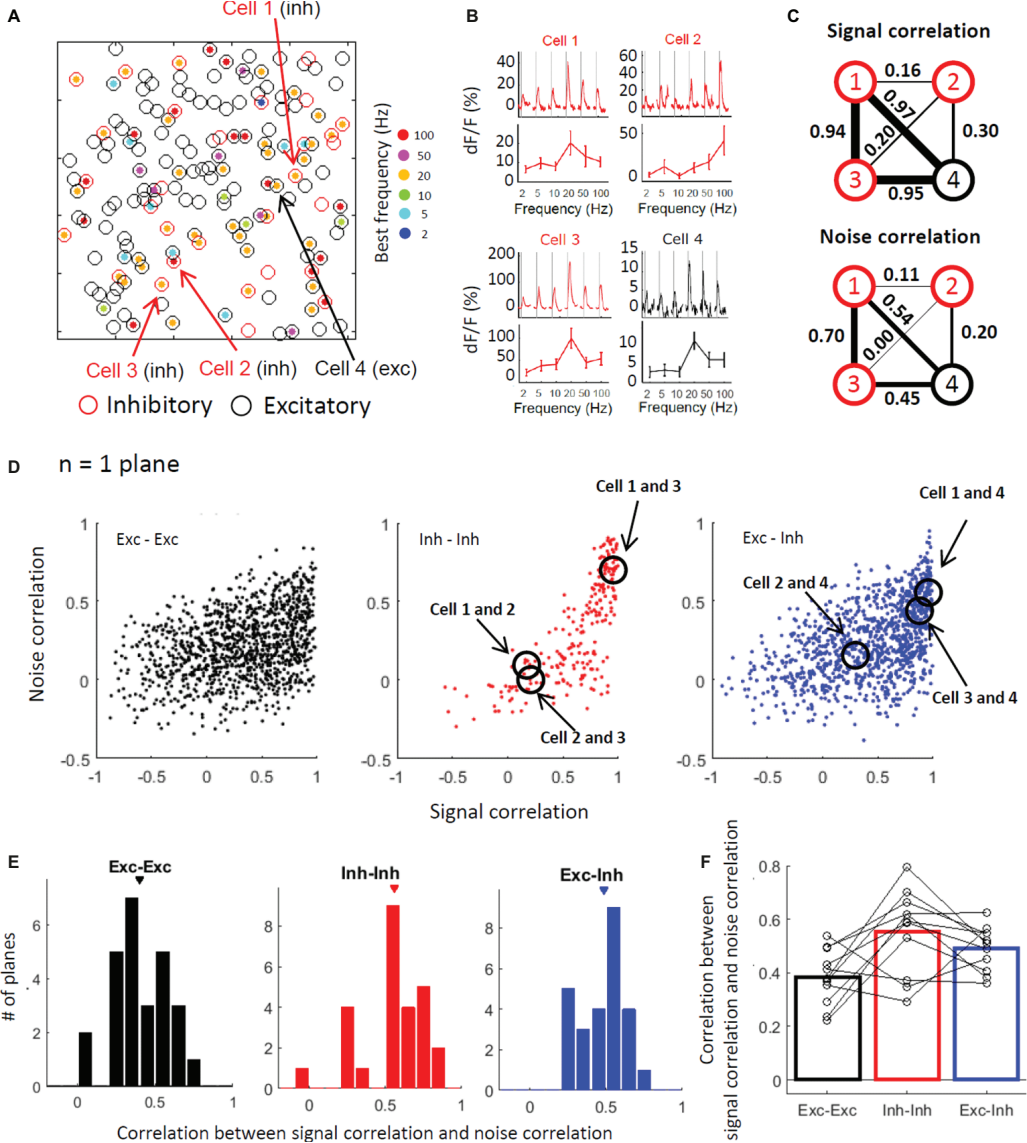
Excitatory and inhibitory cells with similar stimulus selectivity are more active together across trials. **(A)** Spatial distribution of the best frequencies in individual neurons as shown in Figures 4A,C. The best frequencies are indicated with a color code. **(B)** Response time courses (upper panels) and stimulus selectivity curves (lower panels) of three inhibitory cells (cells 1–3) and one excitatory cell (cell 4). The cells’ locations are demonstrated with arrows in **(A)**. **(C)** Schematic representations of signal (top) and noise correlations during the stimulation (bottom) among the four representative cells. Circles indicate individual cells (cells 1–4) and the line width connecting the two cells indicates the correlation between the two cells. **(D)** Noise correlations plotted against the signal correlation in a representative plane. Pearson correlation coefficients between the signal and noise correlations during the stimulation were 0.35 in the excitatory-excitatory pairs (black, left panel), 0.76 in the inhibitory-inhibitory pairs (red dots, center panel), and 0.49 in the excitatory-inhibitory pairs (blue dots). Each dot indicates data from a single pair. **(E,F)** Distribution of the correlation between the signal and noise correlations during the stimulation in all imaged planes (*n* = 26 planes, **E**) and 11 mice **(F)**. **(E)** The correlation was computed in each imaged plane. Exc-Exc: 0.40 ± 0.03 (mean ± SE), Inh-Inh: 0.56 ± 0.04, and Exc-Inh: 0.48 ± 0.03. Triangles indicate the mean values. **(F)** The correlations were averaged in individual animals (open circles). The bars represent the mean of 11 mice. Exc-Exc: 0.38 ± 0.03, Inh-Inh: 0.56 ± 0.05, and Exc-Inh: 0.49 ± 0.02. Exc-Exc: excitatory pairs, Inh-Inh: inhibitory pairs, and Exc-Inh: excitatory and inhibitory pairs.

Finally, we explored the correlation between the signal and noise correlations during stimulation. Figure 6A shows a plane map depicting positions of the individual cells and their best frequencies. Figure 6B shows the response time courses of the 3 inhibitory cells (cells 1–3) and one excitatory cell (cell 4) depicted in Figure 6A. Among the 3 inhibitory cells, cell 1 and cell 3 have similar stimulus selectivity (i.e., high signal correlation), while cell 2 has a relatively different selectivity. Although inhibitory cells tend to have similar selectivity, there is still variability in the response properties of inhibitory cells within a plane as previously depicted (Figures 4A–F). Cell 4, which is the only excitatory cell, has a relatively similar selectivity to that of inhibitory cells 1 and 3. Among these cells, the cells with the higher signal correlation (cells 1, 3, and 4 in the upper panel of Figure 6C) also have higher noise correlations (lower panel in Figure 6C). Figure 6D illustrates the relationship between signal and noise correlations in the representative plane. The cell pairs with the higher signal correlation appear to have a higher noise correlation. This finding indicates that cells with similar selectivity tend to be active together at the point of trial-to-trial variability, implying that there are functional networks among cells with similar selectivity. The higher noise correlation between the cells with the higher signal correlation is confirmed by the positive correlation between the signal and noise correlation in most imaged planes across all pair types (Figure 6E, Exc-Exc: 0.40 ± 0.03, Inh-Inh: 0.56 ± 0.04, and Exc-Inh: 0.48 ± 0.03). In particular, the excitatory-inhibitory pairs and inhibitory-inhibitory pairs showed higher correlations than the excitatory-excitatory pairs across all planes (Figure 6E; all plane comparison, *P* = 0.036 for Exc-Exc vs. Inh-Inh, *P* = 0.012 for Exc-Exc vs. Exc-Inh, and *P* = 0.37 for Inh-Inh vs. Exc-Inh by the signed-rank test with the Bonferroni corrections). This tendency was also observed in the across animal comparisons (Figure 6F; Exc-Exc: 0.38 ± 0.03, Inh-Inh: 0.56 ± 0.05, Exc-Inh 0.49 ± 0.02, *P* = 0.10 for Exc-Exc vs. Inh-Inh, *P* = 0.029 for Exc-Exc vs. Exc-Inh, and *P* = 0.44 for Inh-Inh vs. Exc-Inh by the signed-rank test with the Bonferroni correction). The high correlation between the signal and noise correlations implies that cells with similar selectivity, including both excitatory and inhibitory cells, work together in functional networks.

## Discussion

Although the barrel area of the rodent S1 has been extensively studied, sensory processing in S1 outside the barrel area has not been well understood. Using two-photon *in vivo* calcium imaging, we recorded the responses of excitatory and inhibitory cells to vibro-tactile stimuli in the mouse S1 hind limb area. The best stimulus frequency (i.e., the stimulus that evoked the largest response from a neuron) of both the excitatory and inhibitory neurons covered all vibro-tactile frequencies from low (2 Hz) to high (100 Hz) frequencies (Figure 2). The excitatory cells responded to fewer frequency stimuli, while the inhibitory cells were more responsive (Figures 3A,B) and less selective (Figures 3C–F) than the excitatory cells. Spatial arrangements of both types of neurons for stimulus selectivity were found to be intermingled in a “salt and pepper” fashion (Figures 4A,C,I). The stimulus selectivity of the inhibitory cell pairs tended to be more similar than those of the excitatory pairs in a local population (Figures 4E,F ), and the selectivity of the individual inhibitory cells was positively correlated with the averaged selectivity of the excitatory cells (Figures 4G,H). Furthermore, noise correlations tended to be higher in the inhibitory cell pairs than those in the excitatory cell pairs (Figures 5A,B). Signal correlations were positively correlated with noise correlation, especially in the excitatory-inhibitory cell pairs and inhibitory-inhibitory cell pairs (Figure 6). Based on these findings, we suggest that excitatory cells represent information about a specific stimulus and also that excitatory and inhibitory cells with similar selectivity work together as a functionally connected network. This network may be useful for the representation of vibro-tactile frequencies in the S1 hind limb area.

### Vibro-tactile Stimulus Representation in the S1 Hind Limb Area

All six applied vibro-tactile frequencies were able to evoke responses from a proportion of cells in both cell types, and the best stimulus frequencies were distributed in all six frequencies (Figure 2). The proportion of responsive cells tended to be higher among the inhibitory cells compared to that among the excitatory cells (Figure 3A). The higher response rate of inhibitory cells and the lower response rate of excitatory cells are consistent with previous reports investigating the barrel area (Gentet et al., 2010; Crochet et al., 2011; Peron et al., 2015). In the barrel area, the lower activity of excitatory neurons likely results from inhibitory synaptic inputs (Crochet et al., 2011). This hypothesis has been supported by a report showing that the suppression of inhibitory cells leads excitatory cells to fire more action potentials (Gentet et al., 2012). The lower activity of excitatory cells in the hind limb area may also be due to the strong inhibition by neighboring inhibitory cells.

We also found that the excitatory cells responded to a fewer number of stimulus frequencies and showed higher selectivity than the inhibitory cells. It can be assumed that the excitatory cells in the S1 hind limb area represent information related to a narrow stimulus range at the single cell level and cover all stimulus frequencies used in this study collectively as a population. In previous studies investigating the rat barrel cortex, cortical cells were found to encode whisker deflection velocity and vibration speed (Pinto et al., 2000; Arabzadeh et al., 2004; Gerdjikov et al., 2010). Although our results did not reveal specific stimulus parameters encoded in the cortical cells (e.g., frequency, amplitude, velocity, etc.), our results suggest explicit representation of vibro-tactile stimuli specifically in excitatory cells.

We also explored the spatial arrangement of the best frequencies (Figures 4A,C,I). The signal correlation of any cell pair type remained virtually constant independent of the distances between the cells (Figure 4I); there is no obvious relationship in which closer cells share similar stimulus selectivity, and the spatial arrangements of the selectivity is “salt and pepper”-like. This finding is consistent with previous studies investigating the barrel cortex, which have also found that the whisker tunings are highly heterogeneous among neighboring neurons (Kerr et al., 2007; Sato et al., 2007; Clancy et al., 2015; but also see Andermann and Moore, 2006; Kremer et al., 2011; Peron et al., 2015; Pluta et al., 2017).

### Broader Tuning of Inhibitory Cells for Vibro-tactile Stimuli in the Mouse S1 Hind Limb Area

In the rodent primary visual cortex, neurons are known to be selective to the orientation and direction of a moving stimulus, whereas L2/3 inhibitory cells have been reported to be less selective than excitatory cells (Sohya et al., 2007; Liu et al., 2009; Kerlin et al., 2010; Runyan et al., 2010; Hofer et al., 2011; Atallah et al., 2012). The broader tuning of inhibitory cells has also been reported in the auditory cortex (Wu et al., 2008; Sakata and Harris, 2009; Sun et al., 2010; Moore and Wehr, 2013; but also see Dorrn et al., 2010), the entorhinal cortex (Buetfering et al., 2014), olfactory bulb (Kato et al., 2013; Miyamichi et al., 2013), and the hippocampus (Hu et al., 2014). In the S1 barrel cortex, it has also been reported that fast-spiking units, that is, putative inhibitory cells, exhibit poor direction selectivity compared to the regularly spiking units, that is, putative excitatory cells (Bruno and Simons, 2002; Kida et al., 2005). However, to the best of our knowledge, there are no reports on response selectivity of genetically identified excitatory and inhibitory cells to cutaneous vibro-tactile stimuli with multiple frequencies in the S1. Here, we demonstrated that genetically identified inhibitory cells are relatively less selective to vibro-tactile stimuli, responding to more stimulus frequencies compared to excitatory cells, which is consistent with other sensory areas (Figures 2,**3**). The broader tuning of the inhibitory cells has been viewed to reflect the nonselective integration of excitatory cell inputs (Kerlin et al., 2010; Scholl et al., 2015). This is supported by the relatively dense synaptic connections from the surrounding excitatory cells to the inhibitory cells (Holmgren et al., 2003; Hofer et al., 2011; Avermann et al., 2012).

The broader tuning of inhibitory cells is probably suited for modulating the activities of excitatory cells independent of stimulus types. In the barrel area of mouse S1, sensory responses of the excitatory cells are regulated by inhibitory inputs, with only about 10% of the excitatory cells firing action potentials (Crochet et al., 2011). The broader tuning of inhibitory cells may be useful to achieve this sparse representation. And the broad inhibition across stimuli is likely to sharpen the tuning to a specific stimulus (i.e., increase the stimulus selectivity) by suppressing activities to suboptimal stimuli (Isaacson and Scanziani, 2011; Tremblay et al., 2016 for reviews). The tuning of inhibitory inputs (e.g., broad or sharp tuning across stimuli) is viewed to be one of the important factors that determine the selectivity of the excitatory cells (Isaacson and Scanziani, 2011; Wood et al., 2017 for reviews). The inhibitory cells are also involved in the gain modulation of the excitatory cells’ response. Specifically, normalization, one of the gain modulations in which activity suppression depends on the overall activity of surrounding cells, is often observed in the visual and other cortical areas (see Carandini and Heeger, 2012, for a review).

### Functional Subnetworks Between Excitatory and Inhibitory Cells With Similar Stimulus Selectivity

It has been previously reported that excitatory cells are sparsely connected to each other with weak synapses on average, whereas synaptic interactions between excitatory and inhibitory neurons are dense and strong (Holmgren et al., 2003; Packer and Yuste, 2011; Avermann et al., 2012). Inhibitory cells interact with other inhibitory cells through gap junctions (Galarreta and Hestrin, 1999; Gibson et al., 1999; Tamás et al., 2000; Fukuda et al., 2006) and show correlated spontaneous activity (Hofer et al., 2011; Inoue et al., 2015; Karnani et al., 2016). Our findings are consistent with these previous studies. The stimulus selectivity of inhibitory cells tended to be more similar to each other (Figures 4E,F). Inhibitory-inhibitory cell pairs are more likely to be active together across trials during vibro-tactile stimuli and even when not driven by stimuli (Figures 5A–D). Furthermore, the inhibitory-inhibitory cell pairs that were active together during baseline activity also tended to respond together to the vibro-tactile stimuli across trials (Figures 5E,F), suggesting that intrinsic connections affect stimulus-driven activity. We also found that cell pairs with similar selectivity tended to be active together (Figure 6). Although high noise correlations in neuron pairs with similar tuning have been reported in the barrel area of mouse S1 (Kwon et al., 2017), its relation with cell-type specificity has not been addressed. Our results revealed that this tendency is relatively strong in excitatory-inhibitory and inhibitory-inhibitory cell pairs. These findings imply that cells with similar selectivity, especially inhibitory cells, work together as a functionally connected network. This network of inhibitory cells could be the basis for the modulation of the surrounding cells by inhibitory cells.

Many excitatory-inhibitory cell pairs showed positive signal and noise correlations, and only a small proportion showed negative signal and noise correlations (Figures 4 **,** **5**). We speculate that these positive correlations partly reflect a direct interaction between excitatory and inhibitory cells averaged over several seconds. On the other hand, only a small proportion of excitatory-inhibitory cell pairs show negative correlations, and the inhibitory effects from the inhibitory cells to the excitatory cells were rarely observed. We assume that the effects of the inhibitory cells to the excitatory cells are masked by the excitatory inputs from the excitatory cells to the inhibitory cells and/or shared inputs among excitatory and inhibitory cells. The excitatory and inhibitory cells are often reciprocally connected with each other (Yoshimura and Callaway, 2005; Avermann et al., 2012), and the pair is also likely to receive shared inputs (Yoshimura and Callaway, 2005; Gentet et al., 2010). In excitatory-inhibitory cell pairs, the negative correlation would be caused by inhibitory inputs from the inhibitory cells to the excitatory cells, whereas the positive correlation would be caused by excitatory inputs from the excitatory cells to the inhibitory cells. The positive correlation would also be caused by shared inputs. In the barrel area, nearby excitatory and inhibitory cell pairs tend to show correlated membrane fluctuation *in vivo*, suggesting the strong shared and/or excitatory inputs (Gentet et al., 2010). Therefore, strong shared and/or excitatory inputs may lead many excitatory and inhibitory pairs to the positively correlated activities in general. A slightly higher proportion of the excitatory-inhibitory cell pairs showed negative correlation than the other pairs (Figure 4E). And some of these pairs may reflect the strong inhibitory effects in their interactions. We note that our discussion described above is based on the results obtained from the activities averaged over several seconds. The calcium signals have slow kinetics, and our sampling rate was low (i.e., 30 Hz = ~33 msec sampling interval). Thus, it is impossible to detect the fine temporal dynamics of both excitatory and inhibitory interactions with our methods.

The barrel area has a column-like cytoarchitecture in which each barrel mainly represents input to an individual vibrissa, whereas the hind limb area has no such specific structures. Despite this, we obtained similar response properties in the hind limb area. Putative inhibitory cells have been reported to be broadly tuned in the barrel cortex (Bruno and Simons, 2002; Kida et al., 2005). Also similar to our report (Figure 4I), neighboring neurons exhibit slightly different response properties even in the same barrel (Kerr et al., 2007; Sato et al., 2007; Clancy et al., 2015; but also see Andermann and Moore, 2006; Kremer et al., 2011; Peron et al., 2015; Pluta et al., 2017). Thus, a functional network between excitatory and inhibitory cells may also be observed in the barrel area.

We acknowledge that we did not classify the inhibitory cell population into subtypes. GABAergic inhibitory cells can be divided into three largely nonoverlapping subtypes defined by molecular markers (Lee et al., 2010). A recent study suggests an even more detailed inhibitory cell subtype defined by transcriptomic signatures (Tasic et al., 2016). During ongoing activity, cell pairs of the same subtype are activated simultaneously compared to pairs between different subtypes, suggesting the presence of subtype-specific functional networks (Hofer et al., 2011; Inoue et al., 2015; Karnani et al., 2016). In the mouse visual cortex, it has been reported that the three subtypes have different response properties (Liu et al., 2009; Kerlin et al., 2010; Atallah et al., 2012; Wilson et al., 2012) and that learning induces cell-type specific functional connectivity (Khan et al., 2018). Thus, the three inhibitory subtypes are likely to have different roles in the functional subnetworks. It will be important to examine responses of each subtype to fully understand the role of inhibitory cells in sensory processing in the S1.

We also acknowledge that although we aimed to observe cortical responses to cutaneous vibro-tactile stimuli, other peripheral sensors signaling the proprioceptive senses, such as muscle spindles and joint afferents, may also have been stimulated. Spindle fibers’ activities are modulated by stimulating a muscle with various frequencies in the mouse hind limb (Wilkinson et al., 2012). Proprioceptive signals are integrated into cutaneous signals in the S1 of many animals including rodents (Chapin and Lin, 1984; Hsiao, 2008; Yamada et al., 2016). But it is impossible, with our experimental design, to distinguish cortical responses to the somatosensory sense from those to the proprioceptive sense. In the future, recent advances in cell type/pathway specific manipulation through optogenetics may clear this point.

We recorded the neuronal activities under isoflurane anesthesia. Isoflurane anesthesia changes the balance between excitatory and inhibitory inputs (Haider et al., 2013) and also increases correlated activities compared to an awake condition (Goltstein et al., 2015), although 1% isoflurane (similar to our dose) induces a relatively small degree of correlated activity comparable to that induced by a lower concentration of isoflurane (0.5%) in the mouse barrel area (Lissek et al., 2016). The correlated activity has been reported to change depending on the brain state (Poulet and Petersen, 2008; Goltstein et al., 2015; Lissek et al., 2016). Nevertheless, some aspects of our results have been reported in awake mice: inhibitory cells show higher activity (for barrel cortex; Gentet et al., 2010, 2012), lower stimulus selectivity (for the olfactory bulb; Kato et al., 2013; and for the visual cortex; Polack et al., 2013), and higher noise correlations (for the visual cortex; Khan et al., 2018) than the excitatory cells. In the future, recordings from both anaesthetized and awake conditions are required to reveal the entire structure of the functional network.

In summary, we propose that excitatory and inhibitory cells with similar stimulus selectivity work together as a functionally connected network.

## Data Availability

Data and analysis codes are available from the corresponding author upon reasonable requests.

## Ethics Statement

All experiments were carried out in accordance with the institutional animal welfare guidelines of the Animal Care and Use Committee of Kyushu University, and approved by the Ethical Committee of Kyushu University.

## Author Contributions

AH, TY and KO conceived and designed the study. AH and TY conducted the experiments and analyzed the data. AH, TY and KO wrote the manuscript.

### Conflict of Interest Statement

The authors declare that the research was conducted in the absence of any commercial or financial relationships that could be construed as a potential conflict of interest.
