# Supplementary material for: Cell Type Specific Representation of Vibro-tactile Stimuli in the Mouse Primary Somatosensory Cortex
Source: Front Neural Circuits. 2018 Dec 20;12:109. doi: 10.3389/fncir.2018.00109 (PMC6307530; doi:10.3389/fncir.2018.00109)
Supplement: Supplementary file 1 [file Data_Sheet_1.docx]

Supplementary Material

Cell Type Specific Representation of Vibro-tactile Stimuli in the Mouse Primary Somatosensory Cortex

Ayako Hayashi, Takashi Yoshida*, Kenichi Ohki*

*** Correspondence:** Corresponding Author: Kenichi Ohki, Takashi Yoshida
Email: [kohki@m.u-tokyo.ac.jp](mailto:kohki@m.u-tokyo.ac.jp), [takashiy@m.u-tokyo.ac.jp](mailto:takashiy@m.u-tokyo.ac.jp)

**Supplementary Figure 1.** **Identification of the boundary depth between layer 2/3 and 4 in the S1 hind limb area.**

**(A)** Expression patterns of tdTmato in layer 4 neurons under the control of the scnn1a promotor in the S1 hind limb area of Scnn1a-Ai14 mice. Horizontal dotted lines indicate the pial surface (upper line) and 340µm depth (lower line). tdTomato expressing cells were rarely observed between these two lines. Our data were obtained from depth 160–340µm below the pial surface and likely to be from layer 2/3. The 3D volume (xyz) images were obtained by using two-photon microscopy and maximum intensity projection images to the x (or y) axis are shown here. Volume 1 and 2 were obtained from one mouse and volume 3 was from another mouse.

##

**Supplementary Figure 2.** **Inhibitory cells tend to take longer time than excitatory cells to peak and also to return back to the baseline.**

**(A)** Normalized, averaged waveform for responsive cells. Black indicates excitatory cells and red indicates inhibitory cells. For the calculation of Ca^2+^ response decay time constant, two time-courses were used; (1) the response time-course from the peak point till 14 seconds after stimulus onset and (2) time-course starting at 4 seconds after stimulus onset (i.e. the endpoint of stimulation) till 14 seconds after stimulus onset. These two time-courses were fitted with a single exponential function, independently. The time-course with a better curve fitting was selected for each cell, and its decay time was used for this analysis. Only cells with good curve fitting (R^2^ > 0.9) were included for this analysis. **(B, C)** The distribution of responsive cells’ peak time **(B)** and time constant **(C)** are shown. The time constant was measured from time-course of decayed signal. Upper panel (black): excitatory (Exc) cells. Lower panel (red): inhibitory (inh) cells. Triangles indicate mean values. **(B)** Exc: 2.3 ± 0.08 sec (mean ± s.e.), Inh: 3.7 ± 0.07 sec. P = 4.3 × 10^-25^ by Kolmogorov–Smirnov (KS) test. **(C)** Exc: 3.2 ± 0.48 sec, Inh: 4.7 ± 0.21 sec. P = 2.0 × 10^-19^ by KS test.
